# Design and Testing of BACRA, a Web-Based Tool for Middle Managers at Health Care Facilities to Lead the Search for Solutions to Patient Safety Incidents

**DOI:** 10.2196/jmir.5942

**Published:** 2016-09-27

**Authors:** Irene Carrillo, José Joaquín Mira, Maria Asuncion Vicente, Cesar Fernandez, Mercedes Guilabert, Lena Ferrús, Elena Zavala, Carmen Silvestre, Pastora Pérez-Pérez

**Affiliations:** ^1^ Health Psychology Department Miguel Hernández University Elche Spain; ^2^ Alicante-Sant Joan Health District Consellería de Sanidad Universal y Salud Pública Alicante Spain; ^3^ Systems Engineering and Automation Department Miguel Hernández University Elche Spain; ^4^ Consorci Sanitari Integral, L’Hospitalet de Llobregat Barcelona Spain; ^5^ Donostia University Hospital Donostia Spain; ^6^ Navarra Health Service - Osasunbidea Pamplona Spain; ^7^ Patient Safety Observatory Andalusian Agency for Health Care Quality Seville Spain

**Keywords:** patient safety, risk management, root cause analysis, hospital, primary care, frontline health professionals, middle managers

## Abstract

**Background:**

Lack of time, lack of familiarity with root cause analysis, or suspicion that the reporting may result in negative consequences hinder involvement in the analysis of safety incidents and the search for preventive actions that can improve patient safety.

**Objective:**

The aim was develop a tool that enables hospitals and primary care professionals to immediately analyze the causes of incidents and to propose and implement measures intended to prevent their recurrence.

**Methods:**

The design of the Web-based tool (BACRA) considered research on the barriers for reporting, review of incident analysis tools, and the experience of eight managers from the field of patient safety. BACRA’s design was improved in successive versions (BACRA v1.1 and BACRA v1.2) based on feedback from 86 middle managers. BACRA v1.1 was used by 13 frontline professionals to analyze incidents of safety; 59 professionals used BACRA v1.2 and assessed the respective usefulness and ease of use of both versions.

**Results:**

BACRA contains seven tabs that guide the user through the process of analyzing a safety incident and proposing preventive actions for similar future incidents. BACRA does not identify the person completing each analysis since the password introduced to hide said analysis only is linked to the information concerning the incident and not to any personal data. The tool was used by 72 professionals from hospitals and primary care centers. BACRA v1.2 was assessed more favorably than BACRA v1.1, both in terms of its usefulness (z=2.2, *P*=.03) and its ease of use (z=3.0, *P*=.003).

**Conclusions:**

BACRA helps to analyze incidents of safety and to propose preventive actions. BACRA guarantees anonymity of the analysis and reduces the reluctance of professionals to carry out this task. BACRA is useful and easy to use.

## Introduction

Analyzing the causes of unsafe care helps reduce the number of incidents that may cause harm to patients [1-3]. This is the primary reason that incident reporting systems (IRS) for patient safety exist [4].

### Incident Reporting Systems

Incident reporting systems were introduced into the health care sector in the late 1990s. Similar mechanisms can be found in high-risk sectors, such as the nuclear, railway, and aviation industries. They are essentially mechanisms designed to record critical incidents anonymously [3]. Once analyzed, the information collected leads to improved safety. These IRS systems are usually voluntary, but in some countries or industries they are compulsory.

The Australian Incident Monitoring System, the Sentinel Events Reporting Program, and the New York Patient Occurrence Reporting and Tracking System (NYPORTS) in the United States are three of the first programs designed to learn from incidents [5-7]. In Canada, Europe, and Latin America, IRS designed under similar premises are also in operation, but each system tends to follow different regulations, taxonomies, types of incidents reporting, system management procedures, incentives for reporting, and methods and resources for analyzing information collected [8,9].

Incident reporting systems provide a mechanism to identify risks (in this context how and why patients can be harmed) and surface learning opportunities in different organizations, based on their own experience, toward the objective of reducing the frequency of safety incidents [10]. They can and should also be used to share lessons within and across organizations concerned with improving safety. In the end, such mechanisms are designed to enhance patient safety culture in health care organizations [9].

Incident reporting systems represent a central data collection of incidents with the aim to define fields where action is needed most. These systems are well established, but sometimes they do not succeed in developing an action plan to implement corresponding measures conceived to prevent recurring incidents. The success of IRS requires the involvement of frontline health care providers in the action plan (analyzing causes and proposing solutions related to specific incidents).

In order for IRS to be effective, it is critical to overcome the distrust of professionals who fear the possible consequences of reporting incidents [11]. As well, it is essential to make certain that appropriate procedures and resources for quickly analyzing the causes of incidents are already in place [9] and that systems exist to disseminate the conclusions drawn from incident analysis such that similar incidents among health care professionals may be avoided in the future [10-12].

### Incident Analysis for Patient Safety

Root cause analysis, critical incident analysis, and incident simulations are the most useful techniques for investigating what happened [13-15]. Analyzing reported incidents requires time, knowledge, and confidence in the confidentiality of the use of the information [10,16]. However, this is not always possible because as the number of reported incidents increases, the ability to analyze their causes is reduced. There are other care obligations that frontline personnel prioritize more than incident analysis [11]. This prevents middle managers and professionals who are nearest the incident from always being able to participate in its analysis and to propose alternatives or changes to prevent recurrence [11,17,18].

### Patients’ Needs After an Adverse Event

Patients who have suffered an adverse event (AE) should receive information about what and how the incident occurred, and about the measures adopted to prevent recurrence [19,20]. New tools are needed to make certain that patients are informed promptly of countermeasures following incidents.

### Why This Study

Senior and middle managers have direct responsibility for incidents that can be analyzed and assessed in the interest of preventing recurrence [10,21]. For various reasons, however, they do not always make this happen [22,23].

This study’s objective was to develop a tool that helps middle managers and frontline professionals carry out immediate analysis of the causes of incidents related to patient safety whereby they may propose and implement solutions to prevent recurrence. This tool should provide them with the following: a guarantee that the tool adheres to relevant legal regulations; that it engages middle managers and their teams; that it permits appropriate identification of harmful incidents and near misses, probing their immediate and latent causes; and that it conducts a dynamic and agile analysis of these incidents.

## Methods

**Figure 1:**
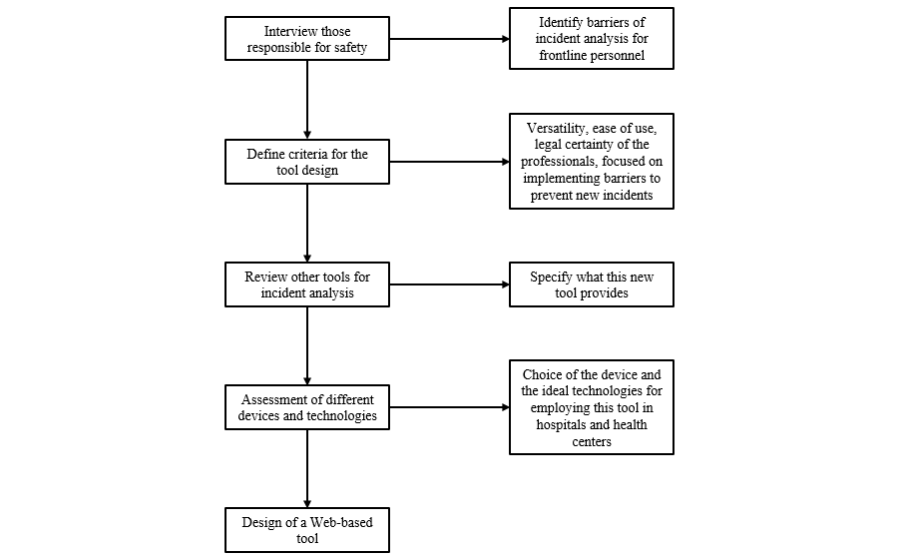
Steps for the design of the incident analysis tool.

Study design of a tool to identify preventive actions for the improvement of patient safety based on the information collected and analyzed from the experiences of previous incidents. Figure 1 describes the steps followed to establish the criteria for this new tool’s design.

### Incident Analysis Tool Requirements

To develop the tool, prior research on the barriers for reporting safety incidents, and incident analysis techniques, were considered first [11,24,25]. This information was expanded by conducting eight semistructured interviews. These interviews were conducted with individuals responsible for safety at hospitals and primary health care centers. They described the difficulties they face in analyzing patient safety incidents and involving professionals and middle managers in the search for solutions to achieve a safer environment for patients.

With this information, a series of criteria were then established with respect to design, navigability, information security and confidentiality, and structure for the analysis of incidents. Based on this, we proposed a feasible projection of what this Web-based tool should deliver in the search for solutions to safety incidents at hospitals and primary care centers.

A review was carried out regarding the tools to conduct an analysis of the causes, consequences, and search for solutions to the incidents, in a broad-based effort to manage risks to patient safety at hospitals and primary care centers. These tools were assessed using criteria established independently by IC, MG, and JJM, with the goal of identifying the characteristics that a new tool should have. From this review, it was determined that the protocol of incident analysis based on the Harvard study [26], the 5 Whys [27], characteristics of root cause analysis, the prioritization of risks employed in failure mode and effect analysis [28], and the matrix employed by the plan-do-check-act (PDCA) tool [29] offered ideal models for this new tool. Due to these characteristics, the tool was renamed BACRA (in Spanish *Basado en Análisis Causa-RAíz meaning* “based on root cause analysis”).

Different alternatives were considered for the development of the incident analysis tool: creating a mobile app for Android and/or iOS tablets and mobile phones (discarded because the necessary devices were not available at most health care centers), developing an executable program (discarded because of the difficulty of installing unofficial apps at health care centers), developing a portable document format (PDF; discarded due to its limitations for generating dynamic content and questions based on previous responses), and a Web form that permitted a sufficient level of flexibility and was accessible from any computer with an Internet connection. The latter was the option chosen.

From this information, a BACRA beta version was developed, a Web tool based on root cause analysis to search for solutions to incidents of patient safety with leadership from middle managers.

### BACRA Design

The tool’s beta version was presented to 43 professionals (middle managers of nursing and surgical medicine, intensive care units, blood banks, laboratories, radiology, mental health, pediatrics, surgery, orthopedics, gynecology, medicine, and primary care). Their feedback helped to improve data access and privacy, and their assessments and suggestions were kept in mind to improve the tool, its user friendliness, and its final result in the form of a summarizing table. In this redesign (BACRA v1.1), fields were added in the solutions table and the app was also personalized to register certain specific types of incidents relevant for hospitals or primary care centers.

BACRA v1.1 was presented to 43 other middle managers, who provided ideas for improving its design (BACRA v1.2). This permitted the elimination of unnecessary information and the introduction of small changes to the types and causes of harm addressed, and allowed the improvement of help texts.

In January 2016, once the tool improvement proposals were introduced, BACRA v1.2 became available to users [30]. It can be used with the main browsers (Chrome, Safari, Microsoft Explorer, Firefox) and operating systems (Windows, OSX, Linux).

### Evaluation of the Web Tool

BACRA v1.1 was used by 13 frontline professionals to analyze distinct types of incidents, whereas its final version (BACRA v1.2) was used by 59 frontline professionals to analyze incidents both with and without harm to patients. Once the analysis was finished, all users had the opportunity to voluntarily assess the Web-based tool’s utility and ease of use.

The evaluation results of both versions were compared using the nonparametric Mann-Whitney *U* test.

### Ethics

This study was approved by the Ethics Committee of Clinical Research at the San Juan de Alicante University Hospital, Alicante, Spain.

## Results

### Bases for BACRA Design

The professionals interviewed pointed out the following main difficulties related to analysis of reported incidents: the lack of time, coupled with the belief that solutions should be proposed by the services responsible for the area of patient safety; the lack of procedures in primary care for addressing this problem; the difficulty in getting middle managers involved in root cause analysis; the delay in communicating analysis incident results; and the legal consequences for professionals. In their opinion, after identifying the type of incident and whether it had caused harm to patients, the tool should help identify causes and consequences in order to gather the basic knowledge necessary to propose solutions intended to prevent recurrence of similar incidents, including a plan of action.

### BACRA Structure

BACRA v1.2 is structured in seven tabs: (1) general information about the tool, (2) hide/show, (3) what consequences did the incident have, (4) when and how did it occur, (5) why did it occur and root of the incident, (6) how could it have been prevented and solutions and plan of action, and (7) printout of the report. A helpline for users who needed guidance was made available. In Textbox 1, the content of each tab is specified.

**Figure 2:**
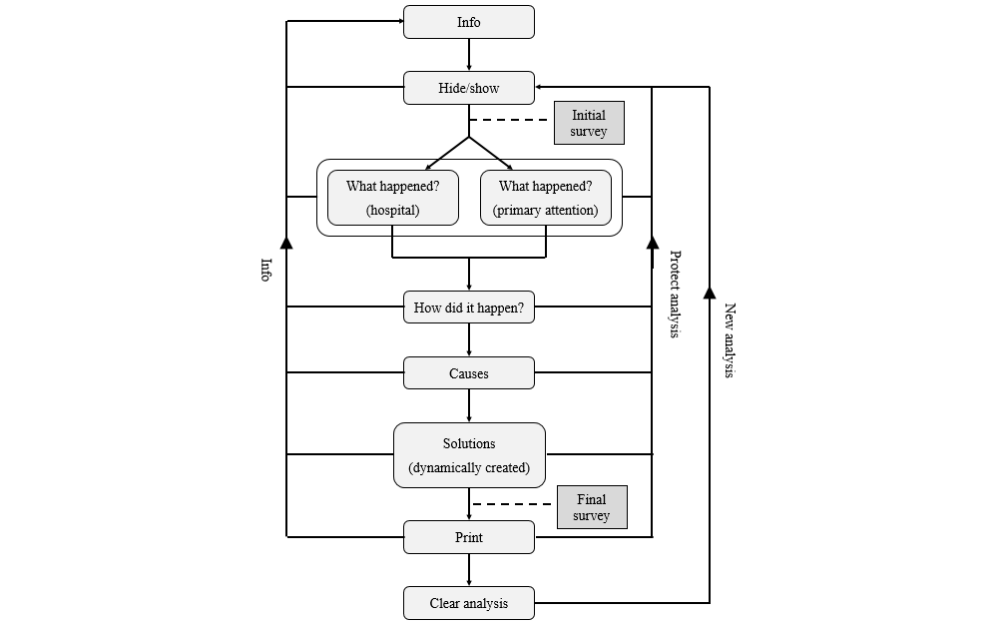
BACRA tool flowchart.

Figure 2 shows a diagram of the app’s different tabs. Moving from top to bottom is the recommended order for the introduction of information, but it is possible to move freely between the various tabs in any order. Each tab’s contents corresponds to the sections described in Table 1.

The information tab is always accessible from any point in the analysis (found at the far left, labeled “Info”; see Figure 2). Withdrawing the analysis is also possible at any time by opening the “Hide/Show” tab and clicking “restore” (erases all data) (see the ascending path of “protect analysis” on the right). Once the analysis is finished, and the reports have been printed, erasing all data is recommended (ascending “new analysis” path on the right).

Before beginning a new analysis, and in order to ensure confidentiality when computers are shared, each user can create a unique password on the “Hide/Show” page (password access; only known to him/her) that will allow access to the analysis in progress for a specific incident. This password is not linked to the person conducting the analysis, but rather to the incident in question. Therefore, the app does not include personalized access whereby the user can consult his/her incidents in progress; instead, this person must generate a new password for each incident they wish to analyze. This way, complete confidentiality of the person conducting the analysis is ensured, protecting the professional and reducing the distrust toward these types of systems. The type of center, either hospital or primary care, must be indicated on the “What Happened?” page. Depending on the choice of center (hospital or primary care), the screen will contain information specific to the type chosen. In addition, the “Solutions” page is dynamically generated with the data introduced in the preceding steps.

**Figure 3:**
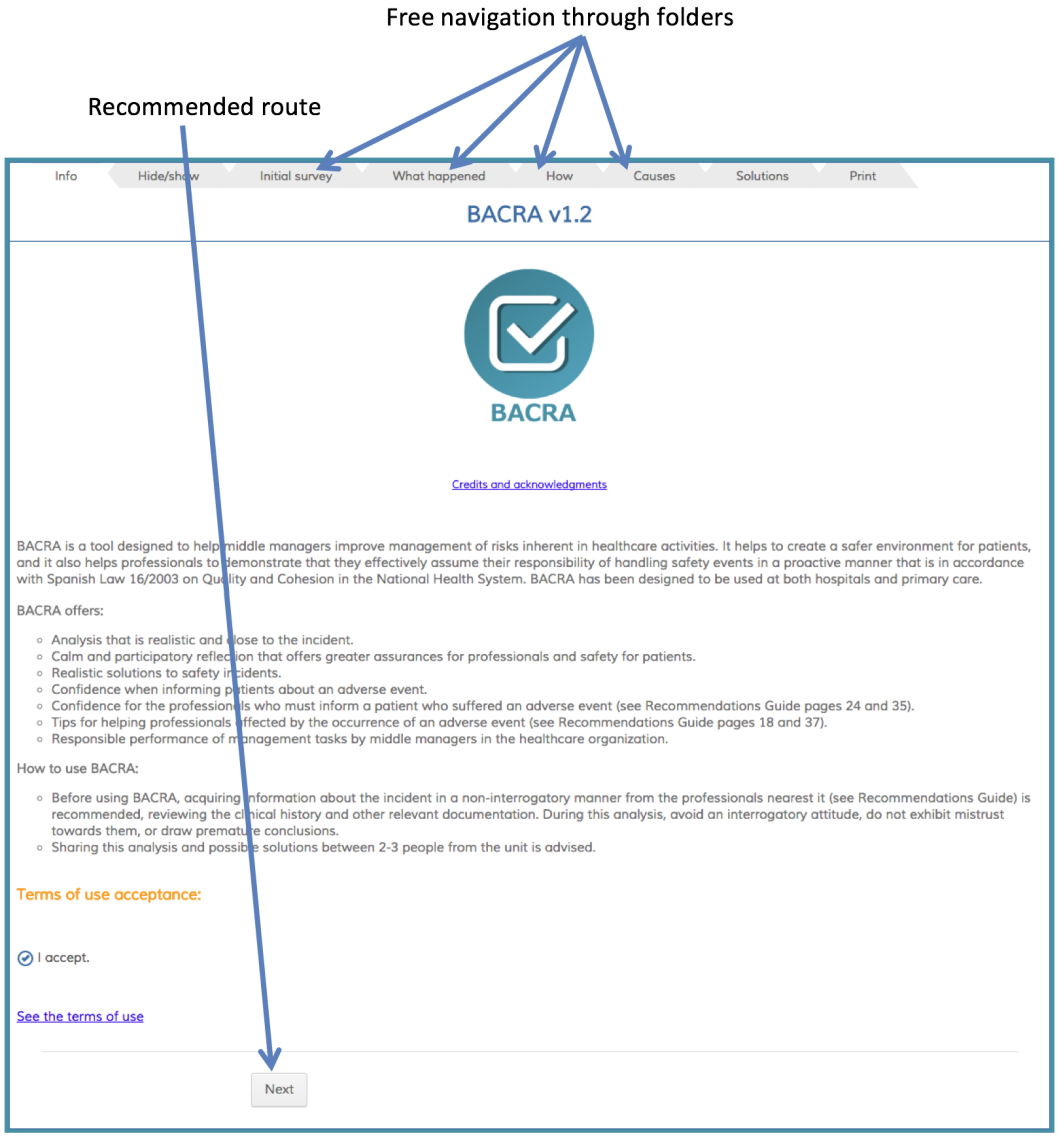
Home page and navigation modes.

Figure 3 shows the home page, where information on using the app is offered. The recommended route for introducing information is visible, but navigating freely between any tab is possible.

**Figure 4:**
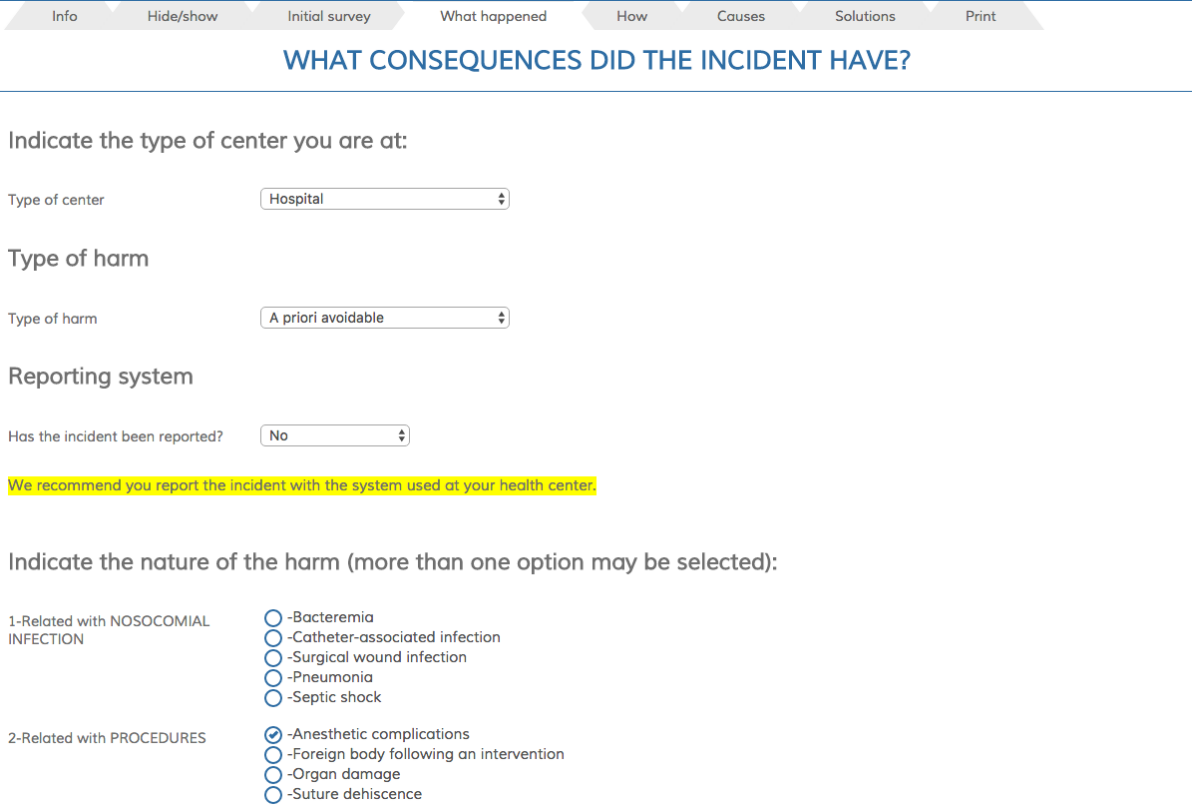
Screenshot of “Consequence” page: type and nature of harm.

Figure 4 shows the manner in which the initial information about the type and nature of the harm is introduced. A list of possible incidents is shown, organized by categories in order to be located easily. The list is specific to the type of center where the analysis is being made, either hospital or primary care. Any number of desired options can be selected and, furthermore, any type of patient harm that does not appear on the list can be introduced as free text.

**Figure 5:**
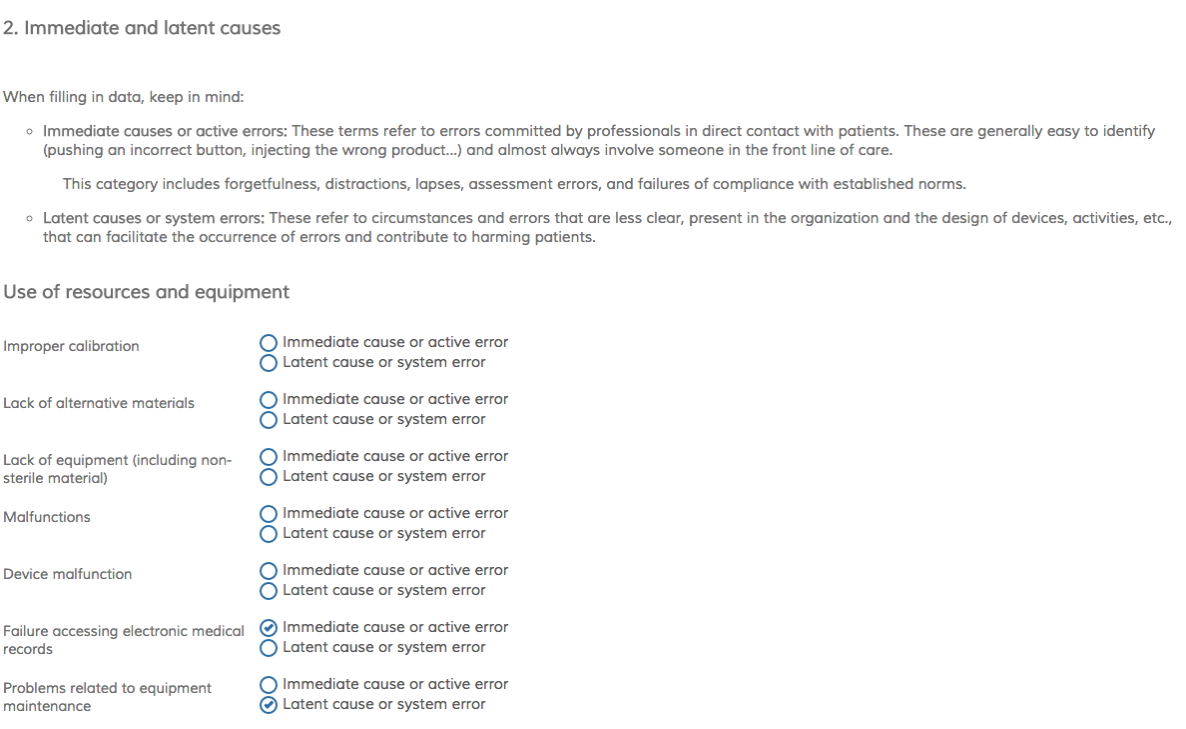
Screenshot of “Causes” page: root of the incident.

Figure 5 corresponds to the “Causes” page. Here, the reasons for the incident and its roots are described. A list of causes is shown, organized by categories. When applicable, additional information may be added, indicating whether the cause is immediate (active error by professionals that is directly related to patients) or latent (system, organization, or device failure).

**Table 1:** Criteria for BACRA to satisfy and analysis of other existing tools.

Criteria	App 1^a^	App 2^b^	App 3^c^	BACRA
Permits incident analysis by a small group (3-5 persons)	Yes	Yes	Yes	Yes
Permits incident analysis in less than 20 minutes	No	No	No	Yes
Uses international taxonomy with help menus in order to correctly interpret the terms	Yes	Yes	Yes	Yes
Permits analysis of adverse events and near errors at hospitals and primary care	Yes	Yes	Yes	Yes
Ensures the privacy and confidentiality of the information	Yes	Yes	Yes	Yes
Offers full guarantees for the legal certainty of the professionals (no data recorded)	No	No	No	Yes
Permits analyzing immediate and latent causes of incidents	No	No	No	Yes
Involves middle managers in the search for solutions	Yes	Yes	No	Yes
Focuses on the search for solutions to prevent recurrence of the same incident	Yes	Yes	No	Yes
Includes how to implement solutions and how to verify whether the anticipated result is obtained	No	No	No	Yes

^a^TPSC Cloud (The Patient Safety Company Cloud).

^b^Sistema de Gestión de Incidentes de Seguridad—Junta de Andalucía.

^c^SiNASP-Sistema de Notificación y Aprendizaje para la Seguridad del Paciente (Learning and Reporting System for Patient Safety).

**BACRA content**

(1) Info: information

- What BACRA is
- What BACRA offers
- How to use BACRA

(2) Hide/Show: hide the form so that nobody else can access it (password access)

(3) What Happened: what consequences did the incident have?

- Care level selection (hospital or primary care)
- Type of harm
- Has the incident been reported?
- Nature of harm
- Related with nosocomial infection
- Related to procedures
- Related with care
- Related with medication
- Others
- Measures adopted with the patient to remedy the harm and to prevent recurrence of another AE related to the first
- Impact (severity and autonomy of the patient)

(4) How: when and how did it occur?

- Date and time of the occurrence and its detection
- Chronology of the facts

(5) Causes: why did this happen? (root of the incident)

- 5 Whys technique
- Immediate and latent causes
- Use of resources and equipment
- Organization and culture of safety
- Factors attributable to professional action
- Intrinsic risk factors for the patient

(6) Solutions: how could it have been avoided? Solutions and plan of action

Risk priority number (RPN)

(7) Print: print report in PDF

**Figure 6:**
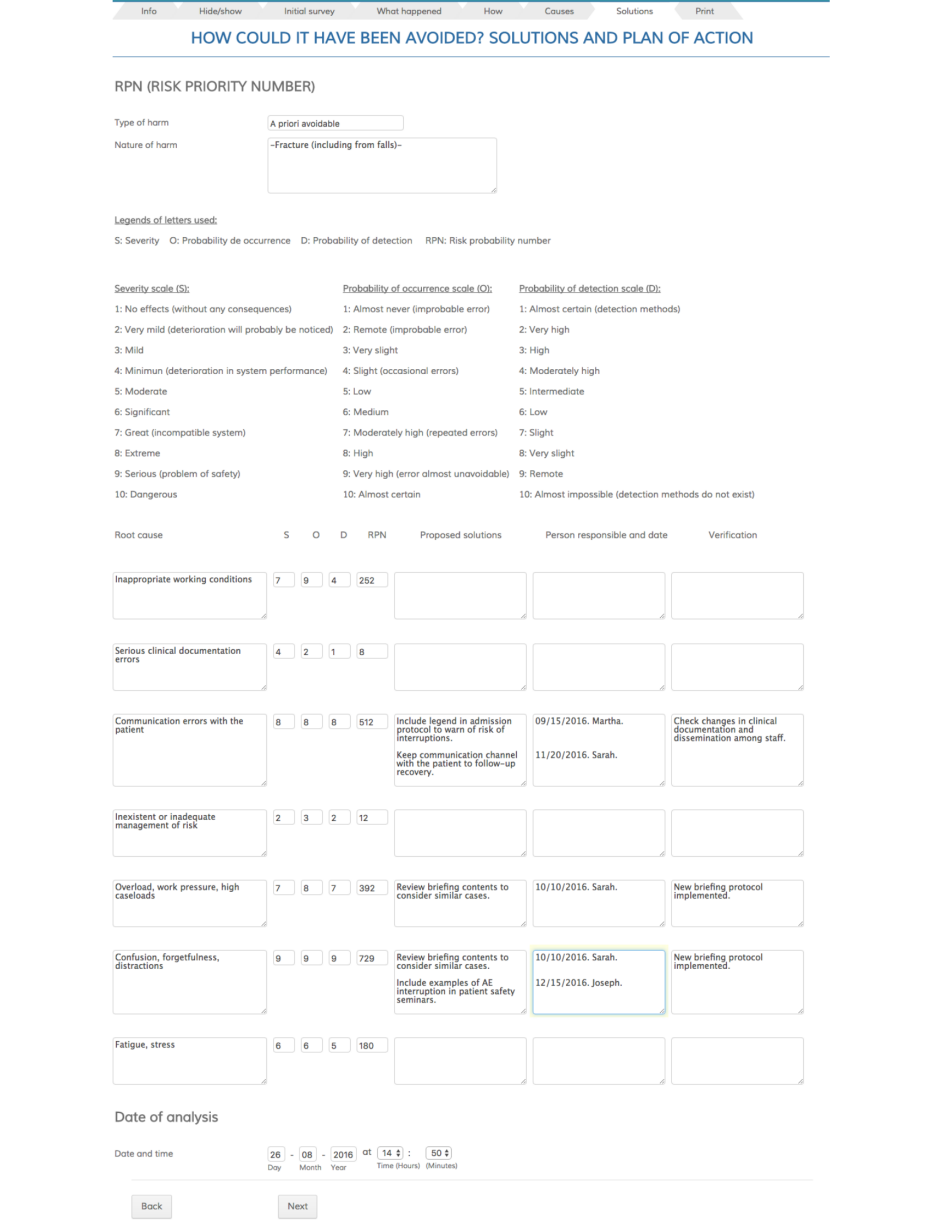
Screenshot of “Solutions” page: final result of the analysis.

Figure 6 shows the final result of the analysis. A portion of the data is generated dynamically based on the information introduced in previous steps. Each of the incident’s causes must be assessed in this tab in terms of their severity, probability of occurrence, and probability of detection. From these values, the app automatically calculates the risk priority number (RPN). For each cause, it asks for solutions to be proposed, which must have an associated date of completion, a professional responsible for implementation, and an outcome measure that permits verification that the proposed solution was successful.

The tool’s final screen allows the printing of a final report that contains information that is detailed depending on where the report is destined to go. Specifically, two options exist: a report for the head of the unit, containing information drawn exclusively from the “Solutions” page (the final result of the analysis), or a report for the center’s safety committee, containing additional data from the “What Happened?”, “How?”, and “Causes” pages.

### Comparison With Other Tools.

Table 1 shows BACRA’s main characteristics compared with other tools designed for reporting incidents for posterior analysis.

### Assessment of the Different Versions

BACRA v1.1 was assessed by 12 of 13 professionals who used it (a response rate of 92%) and BACRA v1.2 was assessed by 47 of 59 professionals who used it (a response rate of 80%). Most of these professionals had reported an incident using other reporting systems at their respective health centers (86%, 62/72).

On a scale of zero to five, BACRA v1.2 was rated higher than BACRA v1.1 in both usefulness (v1.1: mean 3.4, SD 1.6; v1.2: mean 4.3, SD 0.9; z=2.2, *P*=.03) and ease of use (v1.1: mean 2.8, SD 1.5; v1.2: mean 4.2, SD 1.0; z=3.0, *P*=.003).

## Discussion

### Principal Results

BACRA v1.2 was designed to encourage the implementation of preventive measures that impede the repetition of incidents producing harm in patients, as well as incidents that have not yet caused harm but might do so in future due to repetition. This Web-based tool was devised to provide an appropriate response to the difficulties described in the literature, as well as those described by frontline professionals, that have made it difficult to analyze incidents of safety and to seeking solutions. This new instrument promotes health care provider involvement, seeking alternatives to avoid repetition of safety incidents. The data obtained by evaluating the tool justify the changes introduced in v1.2, making it a tool that is useful and easy to use.

BACRA v1.2 is a supplement to an existing IRS to support the frontline health care professional to analyze safety incidents and propose actions to prevent the recurrence of similar incidents. BACRA has been designed to overcome factors limiting reporting and reaching consensus on preventive actions. This tool guarantees anonymity of the analysis and reduces the reluctance of professionals to carry out this task.

The benefits of reporting incidents have been well described, and there is broad consensus that reporting helps improve the management of risks inherent in health care, it strengthens the safety culture, and ultimately increases patient safety [4,5,9]. However, as health care professionals report greater numbers of incidents, those responsible for their analysis become overwhelmed, and the capacity to prevent new incidents may be reduced. Moreover, many times incidents that do not cause harm, or that cause harm that is hardly noticeable, are not analyzed in sufficient detail to prevent similar incidents in the future [11]. Patient safety systems achieve better results when frontline professionals and middle managers get directly involved in incident analysis [31], and especially when they are capable of implementing preventive measures promptly and efficiently. BACRA was designed with this in mind.

### Comparison With Previous Studies

Other studies have pointed out certain barriers that make it difficult to report new incidents, thus making it difficult to transform information into action. Among other reasons, health care professionals attribute their own reticence to fear of punitive action, scant familiarity with incident analysis techniques, difficulties in achieving appropriate feedback, and lack of time to report [32-34].

BACRA guarantees anonymity of the analysis (not only of the reporting) and has been designed to assure that middle managers can become more actively involved in proposing preventive actions to avoid the occurrence of new incidents and thus to save patients from suffering avoidable harm. BACRA is used both to analyze real incidents, which caused harm to patients, and to analyze critical incidents that did not cause harm to patients. This tool provides a framework to identify what has occurred and why, enabling caregivers to determine how to resolve issues and to implant barriers to prevent future failures. The BACRA focus is the implementation of an action plan that defines tasks and responsibilities that follow a clear analytical agenda. The aim of BACRA is to improve patient safety, but it also works to enhance the well-being of frontline professionals by contributing to the creation of a safer clinical context.

BACRA’s Web format is in line with the preferences of electronic systems found in other studies for the analysis of incidents [35]. It allows analysis that is quick and close to the incident, thus increasing reliability in the identification of incident causes and enhancing the validity of new proposals to prevent future incidents.

Approaches to, and conditions of, incident analysis for patient safety have been studied extensively. These studies have demonstrated the critical importance of leadership, effective dissemination channels, and the capacity for rapid action as crucial to the execution of incident analysis that results in preventive action and that enables caregivers to draw lessons from prior experience [25]. These critical elements were included from the outset in the BACRA design. As well, the design takes into account the importance of providing legal protections to those professionals who report and analyze incidents based on the observation that many countries do not have apology laws, which could encourage reporting by reducing the likelihood of legal consequences.

This Web-based tool employs specific techniques proposed by other analysis methodologies seeking involvement from middle managers that have been deemed beneficial to patient safety [25]. The effectiveness of these tools for analyzing incidents of safety has been analyzed in other studies [35].

### Relevance of This Study

The causes of most so-called near errors are not usually analyzed, and so they can continue to cause AEs (reaching the patient) in practice. A large number of health care professionals are not familiar with techniques for analyzing safety incidents; their care responsibilities limit the time they have available to carry out such analyses and, in many cases, they are wary of the consequences that could result from being seen involved in the analysis of incidents. BACRA strives to respond to all these limitations by offering a guideline for conducting an analysis focused on reaching a consensus on actions designed to prevent the recurrence of similar incidents.

BACRA should be used as a supplement to an existing IRS, and not as a stand-alone tool. BACRA is a tool designed by and for middle managers and frontline professionals that does not require specific training in patient safety. This new tool proposes a friendly framework to define an action plan and for implementing corresponding countermeasures that involve frontline health care professionals. A combination of top-down and bottom-up approaches helps to engage health care teams as a whole. They know the questions and, in most instances, they have solutions to offer that will implant barriers designed to prevent harmful incidents in future.

### Tool Limitations

This tool’s main limitation is the need for Internet access, which some centers restrict due to security considerations. Response speed problems have also been detected in computers with limited features. To address these limitations in the future, a version of BACRA could be developed that is capable of functioning locally, without access to the Internet.

BACRA is not an incident reporting system and, therefore, should not be used as such. Its focus is centered on identifying preventive actions to avoid the recurrence of incidents that ultimately do harm patients. Sentinel events could require extensive analysis using the root cause technique.

### Study Limitations

This study was conducted with professionals experienced in incident reporting and thus familiar with basic issues of patient safety. Other users could require more time to become familiar with the tool. This study did not consider certain variables that might influence incident analysis, such as safety culture or perception of the efficacy of proposed solutions. The effectiveness of the proposals to avoid the repetition of similar incidents was not analyzed.

### Recommendations for Practice and Research

The safety culture at health centers can be determinant when implementing this tool. Learning from one’s errors is not easy due to questions that are both attitudinal and practical in nature. In order to exploit BACRA’s advantages, and learn from the experience toward the end of improving patient safety, it is important to promote a proactive culture of safety that acknowledges the possibility that professionals may commit errors in the course of providing clinical assistance. Effective use of BACRA also requires a commitment to the exercise of responsible behavior that improves patient safety.

By using BACRA at health centers, those responsible for the area of patient safety, in coordination with the middle managers of different care units, can agree on the destination of the results reports that the tool produces. For example, agreement could be reached, under appropriate conditions of confidentiality, to disseminate the proposals of applicable measures for specific types of incidents, thereby fostering shared learning to avoid future risks to patients.

Future research could examine the degree to which BACRA and similar tools are accepted by professionals, both those who use these tools and those who make changes in their clinical practice based on proposals reached consensually following the use of BACRA. The extent that BACRA contributes to strengthening of the culture of safety at centers could also be analyzed, for example, by determining whether application of this tool results in changes to management of the risks inherent to clinical practice.

## Abbreviations

AE: adverse event
IRS: incident reporting system
NYPORTS: New York Patient Occurrence Reporting and Tracking System
PDCA: plan-do-check-act
PDF: portable document format
RPN: risk priority number
